# Zinc-α2-Glycoprotein Is Associated with Obesity in Chinese People and HFD-Induced Obese Mice

**DOI:** 10.3389/fphys.2018.00062

**Published:** 2018-02-07

**Authors:** Meijuan Liu, Huijuan Zhu, Yufei Dai, Hui Pan, Naishi Li, Linjie Wang, Hongbo Yang, Kemin Yan, Fengying Gong

**Affiliations:** Key Laboratory of Endocrinology of National Health and Family Planning Commission, Department of Endocrinology, Peking Union Medical College Hospital, Chinese Academy of Medical Science and Peking Union Medical College, Beijing, China

**Keywords:** Zinc-α2-glycoprotein (ZAG), obesity, glucose metabolism, white adipose tissue, peroxisome proliferator-activated receptor gamma coactivator 1 alpha (PGC1a)

## Abstract

Zinc-α2-glycoprotein (ZAG) plays an important role in the regulation of body weight, body fat, and glucose metabolism. In this study, we first measured ZAG levels in serum and *ZAG* mRNA levels in subcutaneous white adipose tissue (sWAT) among overweight/obese patients and lean control subjects. Second, we investigated the effects of ZAG administration on the body weight, body fat and glucose metabolism of high-fat diet (HFD)-induced obese ICR mice and the possible mechanisms involved. The results showed that serum ZAG and mRNA levels in sWAT were significantly decreased in overweight/obese patients and that both showed a negative association with body mass index (BMI) and body weight after adjustment for age and sex. Further partial correlation analysis found that *ZAG* mRNA expression was positively related with several WAT browning-related genes, including uncoupling protein 1 (*UCP1*) (*r* = 0.67) and peroxisome proliferator-activated receptor gamma coactivator 1 alpha (*PGC1a*) (*r* = 0.60), in the sWAT of all subjects. Additionally, intraperitoneal injection of a ZAG expression plasmid (5 μg/injection, four times a week) in HFD-induced obese mice for 8 weeks demonstrated that ZAG overexpression significantly decreased body weight and WAT mass, and greatly increased the glucose tolerance of obese mice, as shown by the intraperitoneal glucose tolerance test (IPGTT) and intraperitoneal insulin tolerance test (IPITT). The staining of UCP1-positive adipocytes was significantly stronger in the sWAT of ZAG-treated obese mice than in that of obese control mice. The mRNA and protein levels of PGC1α in sWAT were significantly increased to 2.2- and 5.3-fold, respectively, compared with HFD obese mice, and there was a strong positive correlation between the expression levels of *Zag* and *Pgc1*α in mouse sWAT (*r* = 0.74). A similar phenomenon was also observed in visceral white adipose tissue (vWAT): the mRNA and protein levels of PGC1α were increased to 1.9- and 3.6-fold, respectively, when obese mice were treated with ZAG. In conclusion, ZAG levels in both sWAT and serum are inversely related with BMI and body weight in Chinese subjects. The action of ZAG on body weight, fat mass and glucose metabolism may be realized through activating PGC1α expression in sWAT and vWAT, then promoting WAT browning in obese mice.

## Introduction

Obesity, affecting ~1.4 billion people worldwide, is reaching epidemic proportions (O'Neill and O'Driscoll, 2015). Given the increasing prevalence of obesity and its associated life-threatening comorbidities, including type 2 diabetes, metabolic syndrome and cardiovascular disease, there is an urgent need to explore the pathogenesis of obesity and develop effective treatment or prevention strategies (Harms and Seale, 2013). Therefore, white adipose tissue (WAT), the largest energy storage organ of the human body, has attracted a great deal of attention from researchers worldwide.

Zinc-α2-glycoprotein (ZAG, also known as AZGP1), a newly identified adipokine, was initially shown to be a tumor-derived molecule (Todorov et al., 1998) responsible for loss of fat mass in patients with cancer cachexia through its action as a lipid-mobilizing factor (Hirai et al., 1998). Recent studies including our research have shown that ZAG is not only expressed in certain tumors but also present in both adipose tissue (Gong et al., 2009) and adipocytes (Bao et al., 2005). Importantly, accumulating evidence demonstrates that ZAG plays a pivotal role in the regulation of body weight and of glucose and lipid metabolism (Mracek et al., 2010; Garrido-Sánchez et al., 2012; Yang et al., 2013). In human subjects, serum ZAG (Gong et al., 2009) and *ZAG* mRNA levels in WAT (Mracek et al., 2010) were negatively correlated with body weight, body mass index (BMI), fat mass, waist and hip circumference, and plasma insulin levels (Mracek et al., 2010; Russell and Tisdale, 2010). Animals studies showed that ZAG overexpression in *ob/ob* and high-fat diet (HFD)-induced obese mice led to significant reductions in body weight and fat mass, as well as a decrease in insulin resistance (Gong et al., 2009; Russell and Tisdale, 2010), whereas ZAG-deficient mice gained more body weight and showed a remarked decrease in lipolysis on both standard and lipid-rich dietary regimens (Rolli et al., 2007). Our previous studies on genetic polymorphism further revealed that the rs4215 polymorphism of the *ZAG* gene were significantly related with overweight/obesity in a northern Han Chinese population (Zhu et al., 2012). All of these results suggest that ZAG is closely related to regulation of body weight as well as glucose and lipid metabolism.

It has been reported that ZAG exerted its effect partially by inhibiting lipogenesis, as well as by stimulating lipolysis and lipid utilization (Sanders and Tisdale, 2004; Gong et al., 2009; Russell and Tisdale, 2011). The effects of ZAG were mediated by interaction with the β3-adrenergic receptor (β3-AR) and can be attenuated by the specific β3-AR antagonist SR59230 (Russell et al., 2004). Interestingly, a third type of adipocyte in WAT, named “beige” or “brite” cells, was discovered recently, and studies have demonstrated that those cells can be activated upon pharmacological activation of β3-AR and that they highly express uncoupling protein 1 (UCP1), then dissipate energy (Fisher et al., 2012; Harms and Seale, 2013; Poher et al., 2015). In this context, activating browning of WAT could be a promising therapy for obesity and its related metabolic diseases (Harms and Seale, 2013; Kajimura et al., 2015). Previous studies *in vivo* (Russell and Tisdale, 2012a) and *in vitro* (Sanders and Tisdale, 2004) demonstrated that ZAG could increase UCP1 expression in brown adipose tissue (BAT). However, it is still unclear whether ZAG has any effect on WAT browning.

Therefore, in this study, we aimed to determine ZAG levels in serum and *ZAG* mRNA levels in subcutaneous WAT (sWAT) and to explore its association with obesity-related parameters in lean and overweight/obese subjects. Furthermore, we investigated the effects of ZAG overexpression on body weight, body fat, and glucose metabolism in HFD-induced obese mice, as well as its possible regulatory targets in adipose tissue, liver and skeletal muscle.

## Materials and methods

### Human study

A total of 40 overweight/obese patients (BMI ≥ 24, age 42.8 ± 4.5 y) and 40 lean control subjects (BMI < 24, age 44.6 ± 8.3 y) were recruited among clinical outpatients and the patients of a medical examination center at Peking Union Medical College Hospital. BMI was calculated as body mass divided by the square of height (kg m^−2^). All recruited subjects received physical and clinical examinations, and blood samples were collected after an overnight fast for the biochemical measurements. Fasting insulin was measured by a Siemens Centaur XP system (Siemens, Tarrytown, USA), and the homeostasis model assessment estimate of insulin resistance (HOMA-IR) was calculated as fasting insulin (mU L^−1^) × fasting glucose (mmol L^−1^)/22.5 (Wallace et al., 2004). Serum ZAG concentrations were determined by commercially available human ZAG enzyme-linked immunosorbent assay (ELISA) kits (Biovendor Laboratorni Medicina, Modrice, Czech Republic) according to the manufacturer's instructions. The intra-assay and inter-assay variation coefficients were 3.9 and 6.6%, respectively.

Human sWAT tissue was collected from 40 morbidly obese patients (BMI ≥ 40) or obese patients (35 ≦ BMI < 40) with obesity-related complications who underwent laparoscopic gastric banding surgery and 21 lean control subjects (BMI < 24, age 48.8 ± 11.3 y) who underwent laparoscopic surgery due to various benign diseases. Abdominal sWAT samples were dissected and then frozen immediately in liquid nitrogen for further studies. The study was approved by the ethics committee of Peking Union Medical College Hospital (No. S-364). All participants signed informed documents before participating in the study.

Total RNA extraction from human sWAT tissue and subsequent reverse transcription were performed as described in our previous studies (Zhu et al., 2015, 2016). In brief, total RNA was isolated from sWAT by use of an E.Z.N.A. Total RNA Kit I (Omega, San Diego, USA). Total RNA in a quantity of 0.5 μg was reverse-transcribed with an Omniscript RT Kit (Qiagen, Maryland, USA). PCR amplification was performed using an ABI 7500 PCR instrument (Applied Biosystems, California, USA), and procedure was conducted in duplicate for each gene. The primer sequences of target genes and internal control genes were listed in Supplementary Table 1. The results were calculated as fold changes in Ct-value relative to the control by using the 2^−ΔΔCt^ formula (Livak and Schmittgen, 2001).

### Animals and treatment

Eight-week-old male ICR mice (weighing 27–30 g, *n* = 31, purchased from HFK Bioscience Co. LTD, Beijing, China) were housed at the Institute of Laboratory Animal Sciences, CAMS and PUMC, at an ambient temperature of 22 ± 1°C under a 12/12 h light/dark cycle and fed *ad libitum*. After 1 week of acclimatization, the mice were weighed and then randomly divided into standard food (SF) group (*n* = 10), which received SF (protein 22%, fat 11% and carbohydrates 67%), and HFD group (*n* = 21), which received HFD (protein 42%, fat 41%, and carbohydrates 17%). All animal experimental protocols were carried out in compliance with the standards of the Guide for the Care and Use of Laboratory Animals and approved by the ethics committee of Peking Union Medical College Hospital.

After 8 weeks, HFD-fed mice were weighed and randomly divided into simple HFD group (*n* = 13, HFD+pcDNA3.1(+) control plasmid) and ZAG overexpression group (*n* = 8, HFD+ZAG expression plasmid). Mice in the SF group were also intraperitoneally injected with an equal volume of pcDNA3.1(+) control plasmid (*n* = 10, SF+pcDNA3.1(+) control plasmid). Plasmid transfection of mice was performed as described in our previous study (Gong et al., 2009). In brief, ZAG expression plasmid (5 μg each) or pcDNA3.1(+) negative control plasmid (5 μg each) in 150 μL of OPTI-MEM medium (Invitrogen, Carlsbad, CA, USA) was mixed thoroughly with Lipofectamine 2000 (10 μL, Invitrogen, Carlsbad, CA, USA) in 150 μL of OPTI-MEM medium, and the total 300 μL of mixture was incubated at room temperature for 30 min, then intraperitoneally injected into mice. This injection was performed at a frequency of four times a week for 8 weeks. The body weights of all mice were routinely recorded once a week.

### Intraperitoneal insulin tolerance test (IPITT) and intraperitoneal glucose tolerance test (IPGTT)

At the end of the intervention, the IPITT and the IPGTT were performed. For the IPITT, mice were fasted for 5 h, and then insulin (Humulin R, Lilly, USA, 0.75 U/kg) was administered intraperitoneally; blood glucose was measured in tail vein at 0, 30, 60, 90, and 120 min with a glucometer (Roche, Basel, Switzerland). For the IPGTT, mice were fasted overnight, then 50% glucose was injected intraperitoneally at a dose of 2 g/kg, and blood glucose was measured as in the IPITT. In addition, the area under the curve (AUC) of glucose concentration between 0 and 120 min was calculated for both the IPITT and the IPGTT.

### Collection of tissue samples and measurements biochemical parameters

After 8 weeks of ZAG intervention, WAT (including inguinal (iWAT), mesenteric (mVAT), perirenal (pVAT), and epididymal (eVAT) white adipose tissue) and BAT (interscapular brown adipose tissue) were excised and weighed. Tissue samples from the liver and right hind limb skeletal muscle were also collected. Serum total cholesterol (TC), triglycerides (TG), free fatty acids, low-density lipoprotein cholesterol (LDL-C), high-density lipoprotein cholesterol (HDL-C) and fasting blood glucose levels were determined by routine automated laboratory methods, and fasting serum insulin was determined by a commercially available mouse insulin ELISA kit (Linco Research Inc., MO, USA). The intra-assay coefficient of variation for insulin was 5.9%.

### Morphological and immunohistochemical staining of adipose tissue in mice

Hematoxylin and eosin (H&E) staining and UCP1 immunohistochemical staining were performed in mouse iWAT according to standard procedures. A primary rabbit polyclonal antibody against UCP1 (1:200 dilution, Abcam, Cambridge, UK) and a secondary goat anti-rabbit IgG antibody (1:2,000 dilution, ZSGB-BIO, Beijing, China) were used in this process. All sections for UCP1 staining were counter-stained with Harris hematoxylin (Histolab, Gothenburg, Sweden) and observed by light microscopy.

### RNA isolation and reverse transcription quantitative PCR (RT-qPCR) analysis of mouse tissues

Total RNA extraction from mouse WAT, liver, and skeletal muscle tissue and subsequent reverse transcription were performed as described above for human adipose tissue. The primer sequences for the target genes and internal control genes were listed in Supplementary Table 2. The expression levels of target genes in WAT and skeletal muscle were normalized to that of peptidylprolyl isomerase A (PPIA), and the expression levels in the liver were normalized to that of β-actin. The results were expressed as fold changes in Ct-value relative to the control according to the 2^−ΔΔCt^ formula (Livak and Schmittgen, 2001).

### Western blot analysis

Total proteins were extracted from mouse iWAT and pVAT using a Total Protein Extraction kit (Applygen, Beijing, China), and the protein concentrations were measured with a BCA protein assay reagent kit (Applygen, Beijing, China). Approximately 30 μg of protein was separated on 10% SDS-PAGE gels and transferred onto nitrocellulose membranes (Millipore, Billerica, MA, USA) through a wet transfer (BIO-RAD, California, USA). The membranes were incubated with the primary antibodies (anti-UCP1, Proteintech, Wuhan, China; anti-PGC1α, Proteintech, Wuhan, China; anti-ZAG, Santa Cruz, Dallas, USA; anti-β-actin, CST, Danvers, MA, USA; anti-GAPDH, CST, Danvers, MA, USA) at a 1:100 to 1:1,000 dilution at 4°C overnight, followed by incubation with a goat anti-mouse/rabbit secondary antibody at a 1:5,000 dilution for 1 h (ZSGB-BIO, Beijing, China). The specific protein bands were visualized by enhanced chemiluminescence (Applygen, Beijing, China). The protein bands were quantified by Quantity One software (Version 4.6.9, BIO-RAD, California, USA). The expression levels of target proteins in iWAT were normalized to that of β-actin, and the expression levels in pVAT were normalized to that of GAPDH.

### Statistical analysis

All data are shown as the mean ± s.e.m. except where it is indicated otherwise. Normal distribution of the data was tested using Shapiro–Wilk test. Non-normally distributed parameters were logarithmically transformed to a normal distribution. One-way ANOVA and Dunnett's T3 *post-hoc* test were used to analyze the differences between groups. Pearson and partial correlation coefficients were used to determine the linear association between ZAG and other parameters. All statistical computations were performed in SPSS 20.0 for Windows (SPSS Inc, Chicago, IL, USA), *P* < 0.05 was considered statistically significant.

## Results

### Serum ZAG levels and other characteristics in overweight/obese patients and normal controls

As displayed in Table 1, body weight, BMI, waist circumferences, fat mass, fasting glucose, fasting insulin, HOMA-IR, and diastolic blood pressure (DBP) were significantly higher in overweight/obese patients than in normal controls. However, serum ZAG levels in overweight/obese patients were significantly decreased by 23.6% compared with those in normal controls. Further association analysis found that serum ZAG levels were negatively correlated with BMI (*r* = −0.336), body weight (*r* = −0.224) and fat mass (*r* = −0.407) after adjusting for age and sex in all subjects.

**Table 1:** General clinical and biochemical characteristics of overweight/obese patients and normal controls.

	**Normal controls (*n* = 40)**	**Overweight/obese (*n* = 40)**	***P*-value**
Age (years)	44.6 ± 8.3	42.8 ± 4.5	ns
Gender (male/female)	20/20	20/20	ns
Weight (kg)	61.2 ± 9.1	77.0 ± 9.7	<0.01
BMI (kg m^−2^)	22.5 ± 2.6	28.5 ± 2.2	<0.01
Waist circumferences (cm)			
Male	83.8 ± 9.2	99.6 ± 10.4	<0.01
Female	76.7 ± 6.8	89.5 ± 5.8	<0.01
Fat mass (kg)			
Male	21.3 ± 6.1	30.6 ± 5.9	<0.01
Female	28.6 ± 4.9	40.0 ± 4.3	<0.01
Fasting glucose (mmol L^−1^)	5.5 ± 1.1	6.0 ± 0.9	<0.05
Fasting insulin (mIU mL^−1^)	6.2 ± 3.5	10.5 ± 6.9	<0.01
HOMA-IR	1.7 ± 1.0	3.0 ± 2.6	<0.05
ALT (U L^−1^)	24.6 ± 10.2	28.4 ± 11.0	ns
AST (U L^−1^)	21.2 ± 10.1	20.8 ± 5.3	ns
BUN (mmol L^−1^)	5.1 ± 1.2	5.0 ± 1.4	ns
Creatinine (μmol L^−1^)	73.1 ± 23.8	82.0 ± 26.4	ns
Uric acid (μmol L^−1^)	252.2 ± 98.6	289.4 ± 96.4	ns
SBP (mmHg)	113.8 ± 19.3	120.3 ± 13.4	ns
DBP (mmHg)	76.0 ± 12.5	81.9 ± 9.5	<0.05
TC (mmol L^−1^)	4.5 ± 1.1	4.8 ± 0.8	ns
TG (mmol L^−1^)	2.1 ± 2.8	2.6 ± 2.0	ns
HDL-C(mmol L^−1^)	1.9 ± 0.5	1.3 ± 0.4	ns
LDL-C (mmol L^−1^)	2.7 ± 0.7	3.0 ± 0.7	ns
ZAG (μg mL^−1^)	50.1 ± 9.0	38.3 ± 11.4	<0.01

*BMI, body mass index; HOMA-IR, homeostasis model assessment estimate of insulin resistance; ALT, alanine transaminase; AST, aspartate transaminase; BUN, blood urea nitrogen; SBP, systolic blood pressure; DBP, diastolic blood pressure; TC, total cholesterol; TG, triglycerides; HDL-C, high-density lipoprotein cholesterol; LDL-C, low-density lipoprotein cholesterol; ZAG, zinc-α2-glycoprotein. Data are shown as the mean ± s.d*.

### The mRNA expression of *ZAG* and WAT browning-related genes in the sWAT of obese patients and normal-weight controls

**Figure 1:**
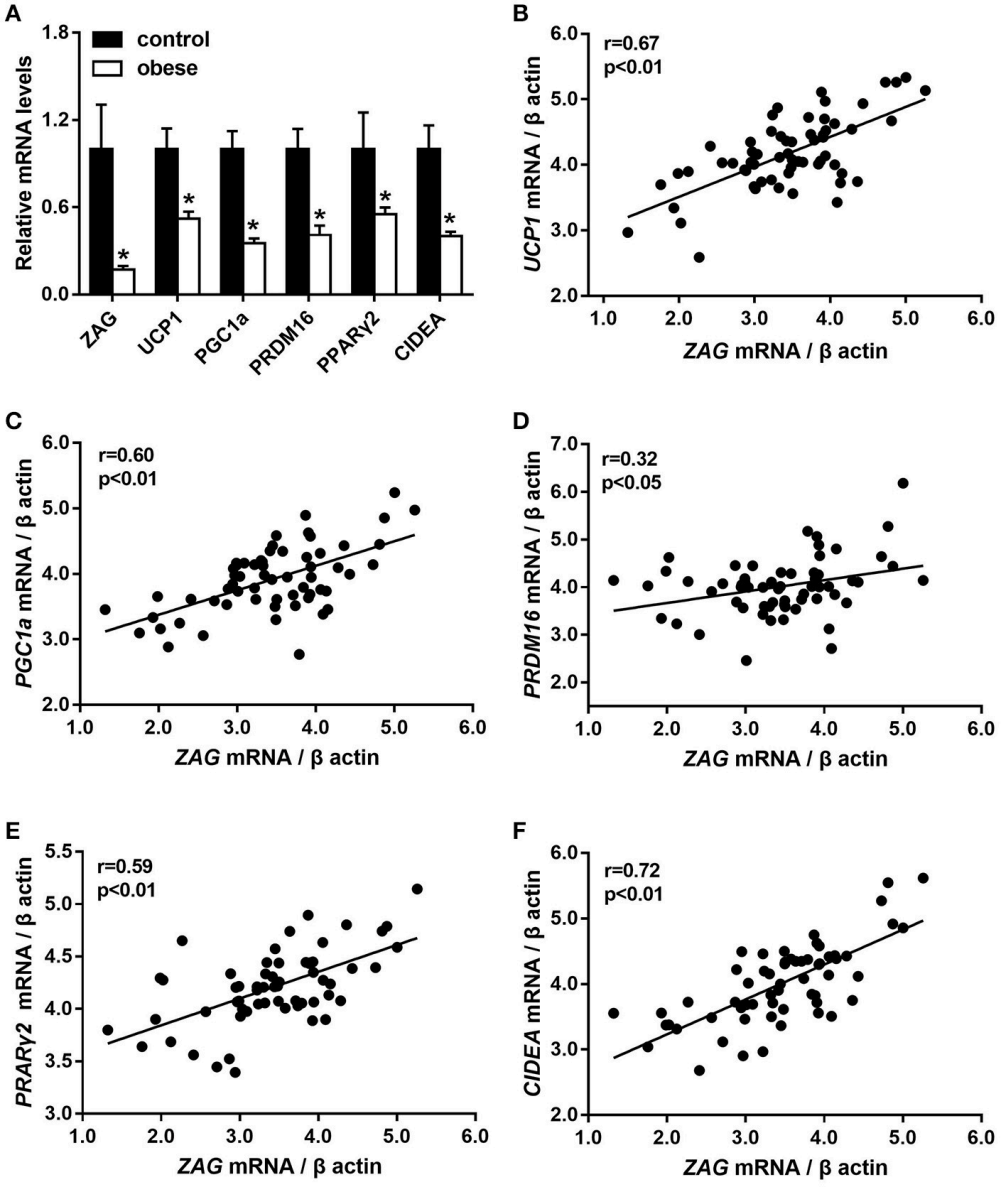
The mRNA levels of *ZAG* and several WAT browning-related genes **(A)** and their correlation analysis **(B–F)** in sWAT of obese patients and normal-weight controls. Partial correlation analysis was performed after adjustment for age, sex, and BMI. ZAG, zinc-α2-glycoprotein; UCP1, uncoupling protein 1; PGC1α, peroxisome proliferator-activated receptor gamma coactivator 1 alpha; CIDEA, cell death-inducing DFFA-like effector a; PRDM16, PR/SET domain 16; PPARγ2, peroxisome proliferator-activated receptor gamma 2. ^*^P < 0.01 vs. normal-weight controls.

As we expected, obese patients had higher body weight, BMI, waist circumference, DBP, and systolic blood pressure (SBP) than normal-weight controls; details are shown in Table 2 (all *P* < 0.05). In accordance with the above serological assay results, *ZAG* mRNA expression in the sWAT of obese patients was significantly decreased by 82.8% in comparison with normal-weight controls (Figure 1A). Partial correlation analysis found that *ZAG* expression had a significant negative correlation with body weight (*r* = −0.417), BMI (*r* = −0.688) and waist circumference (*r* = −0.699) after adjustment for age and sex (Table 3). Moreover, the expression levels of five WAT browning-related genes—*UCP1*, peroxisome proliferator-activated receptor gamma coactivator 1 alpha (*PGC1*α), PR/SET domain 16 (*PRDM16*), peroxisome proliferator-activated receptor gamma 2 (*PPAR*γ*2*), and cell death-inducing DFFA-like effector a (*CIDEA*) were also significantly reduced in obese patients by 48.0, 64.8, 59.1, 44.8, and 59.9%, respectively, compared with the expression in normal-weight controls (all *P* < 0.01, Figure 1A). Further correlation analysis demonstrated that there was a significant positive relationship in mRNA levels between *ZAG* and WAT browning related genes, including *UCP1* (*r* = 0.67), *PGC1*α (*r* = 0.60), *PRDM16* (*r* = 0.32), *CIDEA* (*r* = 0.72), and *PPAR*γ*2* (*r* = 0.59), after adjustment for age, sex and BMI (all *P* < 0.05, Figures 1B–F).

**Table 2:** General clinical and physical examination results of obese patients and normal-weight controls.

	**Normal controls (*n* = 21)**	**Obese patients (*n* = 40)**	***P*-value**
Age (years)	48.8 ± 11.3	29.6 ± 10.0	<0.01
Gender (male/female)	5/16	15/26	ns
Weight circumference (kg)	62.4 ± 5.5	110.4 ± 45.6	<0.01
BMI (kg m^−2^)	22.7 ± 1.4	43.4 ± 8.0	<0.01
Waist (cm)	78.5 ± 7.1	132.9 ± 16.5	<0.01
SBP (mmHg)	108.2 ± 33.7	130.6 ± 35.2	<0.05
DBP (mmHg)	68.0 ± 22.1	83.6 ± 24.5	<0.05

*BMI, body mass index; DBP, diastolic blood pressure; SBP, systolic blood pressure. Data are shown as the mean ± s.d*.

**Table 3:** Correlation between ZAG mRNA expression in sWAT and characteristics of obese patients and normal-weight controls.

	***R***	***P*-value**
Body weight	−0.417	<0.01
Waist circumferences	−0.699	<0.01
BMI	−0.688	<0.01
SBP	−0.072	ns
DBP	−0.079	ns

*sWAT, subcutaneous white adipose tissue; BMI, body mass index; SBP, systolic blood pressure; DBP, diastolic blood pressure; R, correlation coefficient*.

### Effects of ZAG on body weight, body fat mass, biochemical parameters, and insulin sensitivity in HFD-induced obese mice

**Figure 2:**
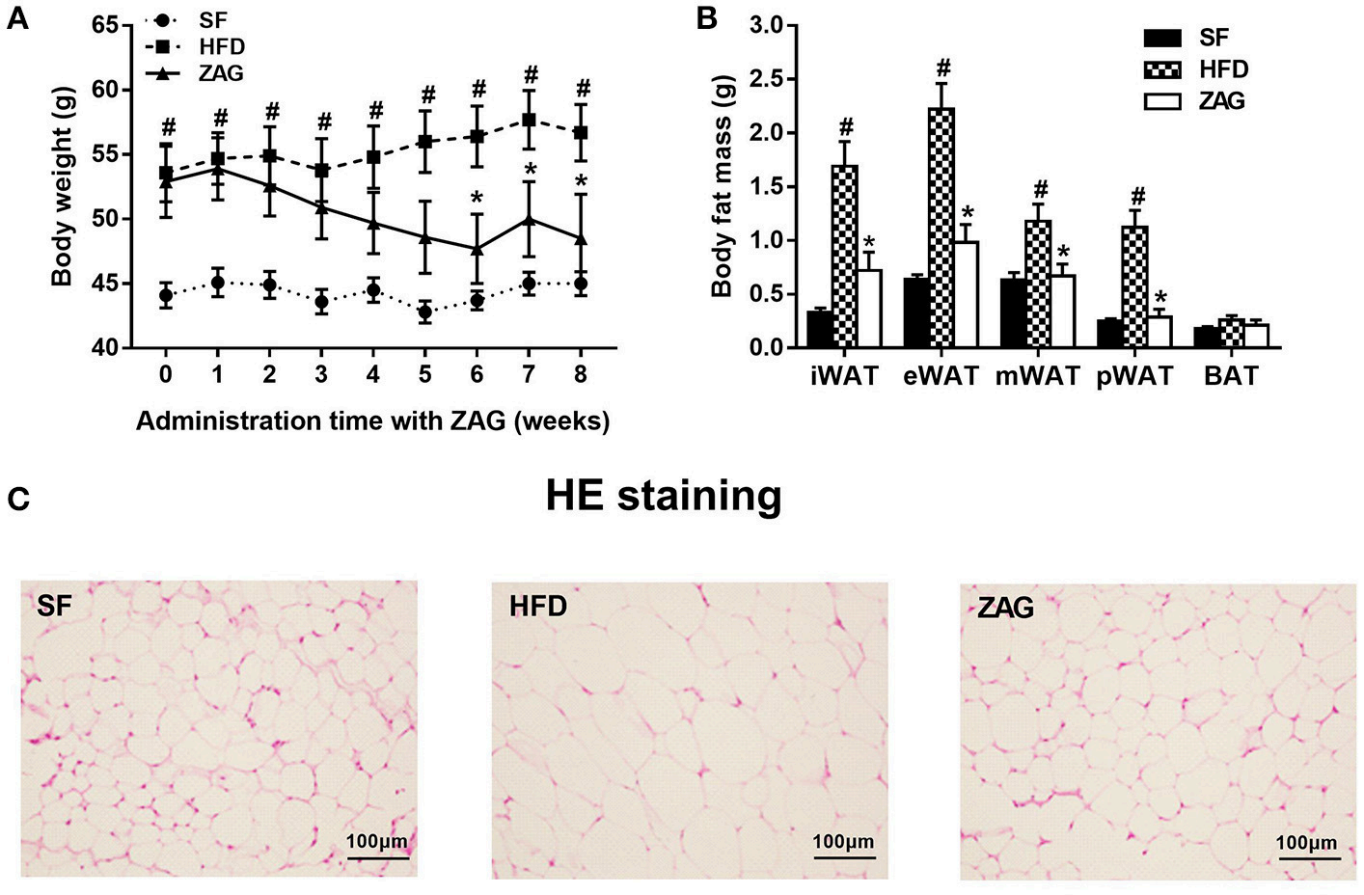
Effects of ZAG overexpression on body weight **(A)** and body fat **(B)** in HFD-induced obese mice. **(C)** Sections of inguinal WAT stained with hematoxylin and eosin. Data are presented as the mean ± s.e.m. SF *n* = 10, HFD *n* = 13, ZAG *n* = 8. iWAT, inguinal white adipose tissue; eVAT, epididymal visceral adipose tissue; mVAT, mesenteric visceral adipose tissue; pVAT, perirenal visceral adipose tissue; BAT, brown adipose tissue. ^#^*P* < 0.05 vs. SF; ^*^P < 0.05 vs. HFD.

After 8 weeks of HFD feeding, an obese mouse model was established successfully as evidenced by significant increased body weight, which reached 1.3 times the weight of mice fed with SF, as shown in Figure 2A. During the period of ZAG intervention, the body weight of obese mice in the HFD group always remained higher than that of SF mice, but gradually reduced after ZAG administration. A significant reduction in body weight was observed at 6 weeks after ZAG treatment (56.4 ± 8.5 g vs. 43.7 ± 2.3 g, *P* < 0.05). At the end of ZAG intervention, the body weight of mice in the ZAG group was reduced by an average of 8.2 g compared with that of the HFD group (Figure 2A). Interestingly, total WAT mass and the percentage of body weight consisting of WAT mass were significantly higher in the HFD group than in the SF group (3.9- and 2.5-fold, respectively), and they significantly decreased by 58.2 and 50.5%, respectively, after 8 weeks of ZAG administration (all *P* < 0.01, Table 4). The same changes were also observed in different deposits of adipose tissue including iWAT, eVAT, mVAT, and pVAT, which were all significantly increased in HFD mice and markedly decreased after ZAG treatment as presented in Figure 2B (all *P* < 0.05). However, no significant change was observed in brown fat mass between these three groups (Figure 2B). Further histological HE staining showed that the adipocyte size of iWAT in HFD mice was notably larger than that in the SF group, while ZAG administration markedly reduced adipocyte size as shown in Figure 2C.

**Table 4:** The effects of ZAG administration on body weight, body fat, and serum biochemical parameters in HFD-induced obese mice.

	**SF (*n* = 10)**	**HFD (*n* = 13)**	**ZAG (*n* = 8)**
Body weight (g)	45.0 ± 0.9	56.8 ± 6.9^d^	48.5 ± 9.7^a^
WAT (g)	1.62 ± 0.13	6.37 ± 0.68^d^	2.66 ± 0.37^b^
WAT (%)	4.70 ± 0.31	11.91 ± 1.01^d^	5.90 ± 0.81^b^
BAT (g)	0.18 ± 0.05	0.26 ± 0.15	0.21 ± 0.15
Fasting glucose (mmol L^−1^)	5.22 ± 0.75	8.00 ± 0.78^c^	5.16 ± 0.92^a^
Fasting insulin (ng mL^−1^)	0.47 ± 0.05	0.52 ± 0.07	0.63 ± 0.13
HOMA-IR	2.15 ± 0.35	4.42 ± 0.88	2.98 ± 1.19
TC (mmol L^−1^)	4.25 ± 0.32	4.90 ± 0.27	4.58 ± 0.36
TG (mmol L^−1^)	0.43 ± 0.05	0.40 ± 0.07	0.64 ± 0.12
HDL-C (mmol L^−1^)	2.23 ± 0.13	2.33 ± 0.12	1.91 ± 0.20
LDL-C (mmol L^−1^)	0.47 ± 0.08	0.54 ± 0.05	0.74 ± 0.09^a^
FFA (μmol L^−1^)	1287.5 ± 109.8	1129.9 ± 82.7	1541.9 ± 98.2^a^
Lipoprotein (a) (mg L^−1^)	4.10 ± 0.53	4.77 ± 0.47	5.38 ± 0.50
UA (μmol L^−1^)	191.90 ± 7.25	195.08 ± 12.85	333.13 ± 23.34^b^

*BMI, body mass index; WAT, white adipose tissue; BAT, brown adipose tissue; TC, total cholesterol; TG, triglycerides; HDL-C, high-density lipoprotein cholesterol; LDL-C, low-density lipoprotein cholesterol; FFA, free fatty acids; UA, uric acid; SF, standard food; HFD, high-fat diet; ZAG, zinc-α2-glycoprotein*.

a *P < 0.05*,

b *P < 0.01 vs. HFD group*;

c *P < 0.05*,

d *P < 0.01 vs. SF group*.

**Figure 3:**
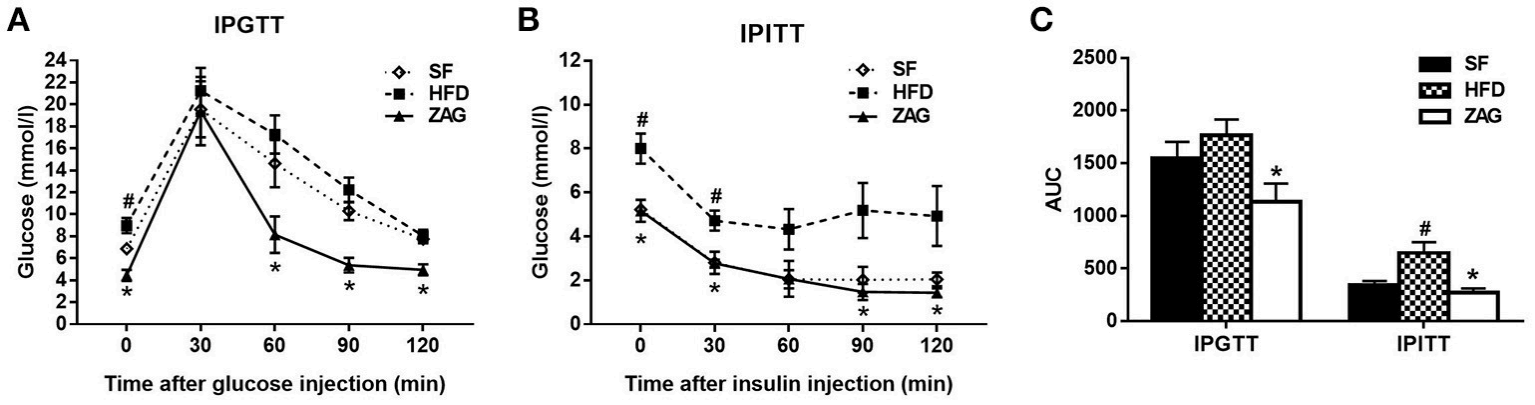
Effects of ZAG overexpression on glucose tolerance **(A)** and insulin sensitivity **(B)** in HFD-induced obese mice. Blood glucose was measured at 0, 30, 60, 90, and 120 min after the injection of glucose (2 g/kg) or insulin (0.75 U/kg); the area under the curve (AUC) was also calculated **(C)**. Data are presented as the mean ± s.e.m. SF *n* = 10, HFD *n* = 13, ZAG *n* = 8. ^#^*P* < 0.05 vs. SF; ^*^P < 0.05 vs. HFD.

The IPGTT showed that the blood glucose levels of ZAG-treated obese mice at 0, 60, 90, and 120 min were significantly reduced by 50.4, 52.8, 56.1, 38.5%, respectively, compared with those of HFD mice (Figure 3A), and the area under the curve (AUC) of glucose also showed a 35.8% decrease in comparison with that in the HFD group (Figure 3C). Furthermore, IPITT also showed a significant reduction in the blood glucose levels of ZAG-treated obese mice at 0, 30, 90, and 120 min by 35.5, 41.3, 71.6, and 71.0%, respectively, in comparison with HFD mice (Figure 3B), and the AUC of glucose also presented a remarkable decrease, falling to 41.8% of the AUC from HFD mice (Figure 3C).

As displayed in Table 4, the fasting glucose levels of HFD-induced obese mice were 53.3% higher than those of SF-fed mice. After these mice were treated with ZAG for 8 weeks, their glucose levels were notably decreased by 35.5% in comparison with those of simple HFD-fed mice. Similar changes were observed in HOMA-IR, which was higher in HFD mice and lower in ZAG-treated mice, although the differences were not statistically significant. In addition, the levels of low-density lipoprotein cholesterol (LDL-C), free fatty acids and uric acid in ZAG-treated mice were all significantly higher than those in the HFD group (all *P* < 0.01).

### Effects of ZAG on the expression of WAT browning-related genes in the iWAT and pVAT of HFD-induced obese mice

**Figure 4:**
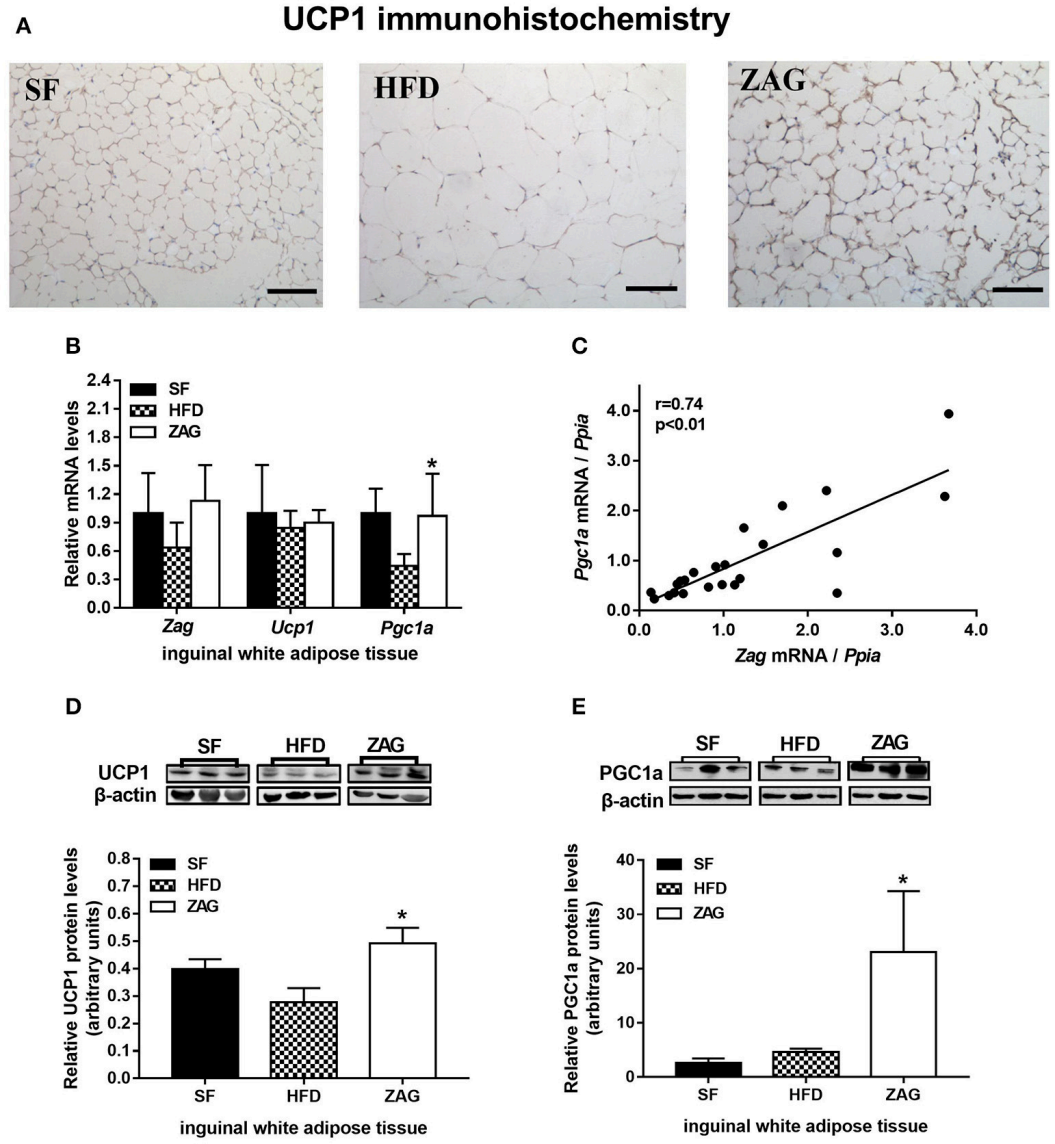
Effects of ZAG on the expression of WAT browning-related genes in iWAT in HFD-induced obese mice. **(A)** Immunostaining for UCP1 in mouse iWAT. **(B)** The mRNA levels of *Zag, Pgc1a*, and *Ucp1* in mouse iWAT. **(C)** Correlation of mRNA levels of *Zag* and *Pgc1a* in mouse iWAT. Spearman's rank correlation analysis was applied to determine the correlation. **(D,E)** The protein levels of UCP1 **(D)** and PGC1a **(E)** in mouse iWAT. The relative integrated density of each protein band was quantified using the program ImageJ, with β-actin as the internal control. Three representative protein band figures are shown. Data are presented as the mean ± s.e.m. SF *n* = 10, HFD *n* = 13, ZAG *n* = 8. iWAT, inguinal white adipose tissue. ZAG, zinc-α2-glycoprotein; UCP1, uncoupling protein 1; PGC1α, peroxisome proliferator-activated receptor gamma coactivator 1 alpha. ^*^P < 0.05 vs. HFD.

As shown in Figure 4A, staining for UCP1-positive adipocytes in the iWAT of HFD-induced obese mice was weaker than that of SF mice, while it was significantly stronger after these mice were treated with ZAG for 8 weeks. In line with these results, the protein levels of UCP1 in the iWAT of ZAG treated mice were significantly increased to 1.8-fold of that of the HFD mice (*P* < 0.05, Figure 4D). In addition, the mRNA and protein levels of PGC1α, widely known as a key regulator that promotes WAT browning, in the iWAT of ZAG treated mice were significantly increased to 2.2 and 5.3 times in HFD mice, respectively, as presented in Figures 4B,E (all *P* < 0.05). Further correlation analysis showed that *Zag* mRNA levels in iWAT were positively related with *Pgc1*α expression (*r* = 0.74) (*P* < 0.05, Figure 4C).

**Figure 5:**
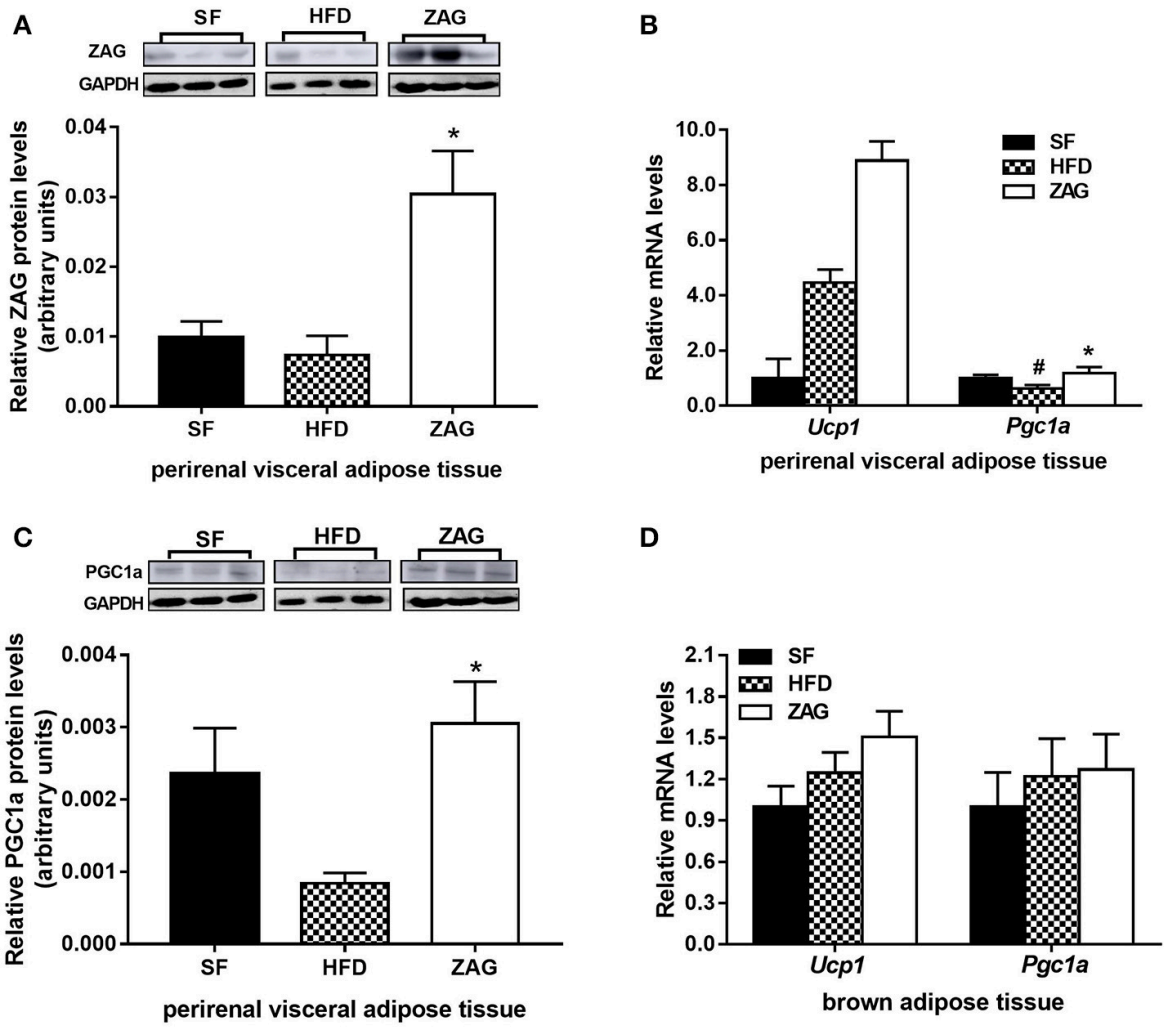
Effects of ZAG on the expression of WAT browning-related genes in pVAT and BAT in HFD-induced obese mice. **(A)** The protein levels of ZAG in mouse pVAT. **(B)** The mRNA levels of *Pgc1a* and *Ucp1* in mouse pVAT. **(C)** The protein levels of PGC1α in mouse pVAT. **(D)** The mRNA levels of *Pgc1a* and *Ucp1* in mouse BAT. For the western blots shown in **(A)** and **(C)**, the relative integrated density of each protein band was quantified using the program ImageJ with GAPDH as the internal control. Three representative protein band figures were shown. Data are presented as the mean ± s.e.m. SF *n* = 10, HFD *n* = 13, ZAG *n* = 8. pVAT, perirenal visceral adipose tissue; BAT, brown adipose tissue; ZAG, zinc-α2-glycoprotein; UCP1, uncoupling protein 1; PGC1α, peroxisome proliferator-activated receptor gamma coactivator 1 alpha. ^#^*P* < 0.05 vs. SF; ^*^P < 0.05 vs. HFD.

As with iWAT, the expressional changes of WAT browning-related genes after ZAG administration were also investigated in pVAT. The ZAG protein level in the pVAT of ZAG-treated mice was markedly increased by 4.13-fold when compared with that of HFD-fed mice (*P* < 0.05, Figure 5A). As shown in Figure 5B, the mRNA level of *Pgc1a* in the pVAT of HFD-induced obese mice was significantly reduced to 62.2% of the value found in SF mice (*P* < 0.05), while it was significantly increased to 1.9 times the value for HFD-induced obese mice after administration of ZAG for 8 weeks (*P* < 0.05). In agreement with the changes in transcription levels, the protein level of PGC1α in the pVAT of obese mice was also significantly increased to 3.6 times the value found in HFD-induced obese mice (Figure 5C, *P* < 0.05). In addition, the mRNA levels of *Ucp1* in pVAT had a tendency to increase after ZAG overexpression (Figure 5B). However, there was no significant difference in *Ucp1* or *Pgc1a* mRNA levels in BAT among these three groups of mice (Figure 5D).

### Effects of ZAG on the expression of glucose-metabolism-related genes in the liver and skeletal muscle of HFD-induced obese mice

**Figure 6:**
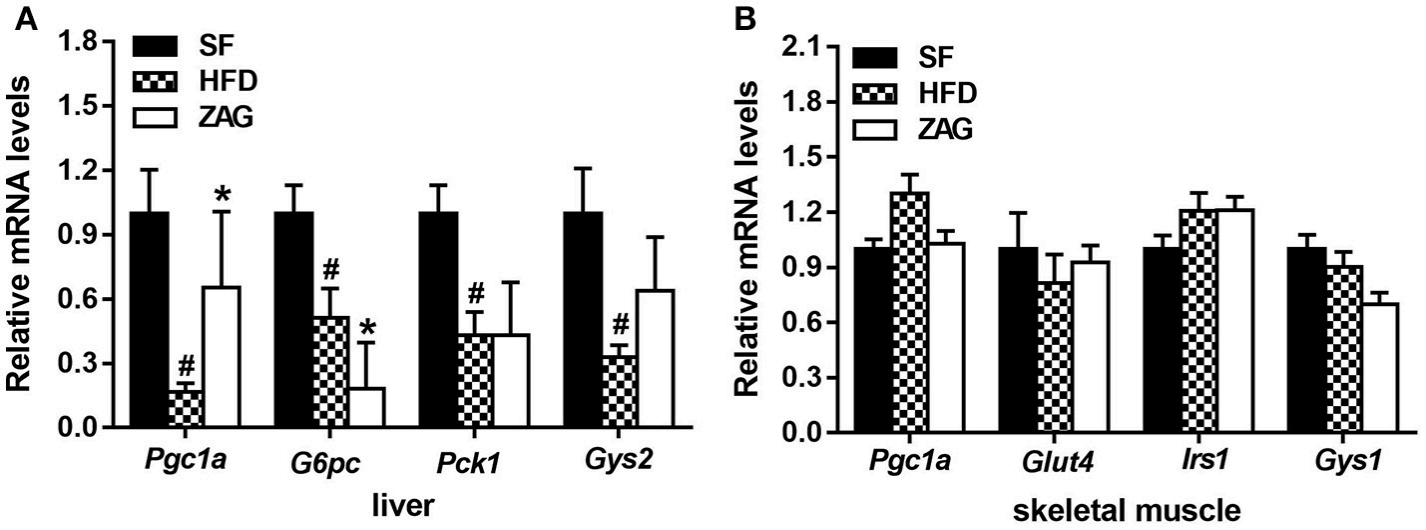
Effects of ZAG on the expression of glucose-metabolism-related genes in the liver and skeletal muscle of HFD-induced obese mice. **(A)** The mRNA levels of *Pgc1a, G6pc, Pck1*, and *Gys2* in mouse liver tissue. **(B)** The mRNA levels of *Pgc1a, Glut4, Irs1* and *Gys1* in mouse skeletal muscle. Data are presented as the mean ± s.e.m. SF *n* = 10, HFD *n* = 13, ZAG *n* = 8. Pgc1α, peroxisome proliferator-activated receptor gamma coactivator 1 alpha; G6pc, glucose-6-phosphatase, catalytic; Pck1, phosphoenolpyruvate carboxykinase 1, cytosolic; Glut4, glucose transporter type 4; Irs1, insulin receptor substrate 1; Gys1, glycogen synthase 1; Gys2, glycogen synthase 2. ^#^*P* < 0.05 vs. SF; ^*^P < 0.05 vs. HFD.

As shown in Figure 6A, the mRNA levels of *Pgc1a*, glucose-6-phosphatase, catalytic (*G6pc*), phosphoenolpyruvate carboxykinase 1, cytosolic (*Pck1*) and glycogen synthase 2 (*Gys2*) in the liver tissue of HFD-induced obese mice were significantly decreased by 83.3, 49.2, 56.7, and 67.3%, respectively, in comparison with that of SF mice (all *P* < 0.05). After treatment of obese mice with ZAG for 8 weeks, the mRNA levels of *Pgc1a* were increased to 3.9 times, while the levels of *G6pc* were notably decreased by 64.1% compared with the HFD-induced obese mice (*P* < 0.05). The mRNA levels of *Gys2* tended to increase after ZAG overexpression (Figure 6A). In addition, the mRNA levels of *Pgc1a*, glucose transporter type 4 (*Glut4*), insulin receptor substrate 1 (*Irs1*) and glycogen synthase 1 (*Gys1*) in skeletal muscle had no significant changes among these three groups as displayed in Figure 6B.

## Discussion

ZAG has recently been reported as a novel adipokine that is expressed at high levels in adipose tissue (Bao et al., 2005). In this study, we found that serum ZAG levels in overweight/obese patients were significantly decreased and had a negative correlation with body weight, BMI and fat mass, consistent with our previous studies (Gong et al., 2009) and previous reports in the literature (Mracek et al., 2010; Russell and Tisdale, 2010). A significant reduction of *ZAG* expression in the sWAT of obese patients was found in subjects of Caucasian origin (Ceperuelo-Mallafré et al., 2009; Selva et al., 2009; Balaz et al., 2014). In the present study, we found that *ZAG* expression in the sWAT of Chinese patients with morbid obesity was also significantly decreased. In addition, our study found a negative relationship between *ZAG* mRNA levels and obesity-related parameters including body weight, waist circumference and BMI, which was consistent with reports by Mracek et al. and by Selva et al. in a Caucasian population (Selva et al., 2009; Mracek et al., 2010). Similar conclusions were also drawn in HFD-induced obese and *ob/ob* mice in our previous research and that of others (Gong et al., 2009; Mracek et al., 2010). Moreover, the relationship between ZAG and obesity was further supported by ZAG administration and ZAG loss-of-function experiments in HFD-induced obese or *ob/ob* mice, which showed that ZAG administration could induce significant reductions in body weight and body fat mass (Russell and Tisdale, 2010), while ZAG knock-out mice gained more weight than normal mice fed with either standard food or lipid-rich food (Rolli et al., 2007). All these findings, together with our previous results demonstrating that the *ZAG* rs4215 SNP was significantly associated with obesity susceptibility in a northern Han Chinese population (Zhu et al., 2012), suggest that ZAG is closely linked to obesity in both humans and mice and that it plays an important role in the regulation of body weight and body fat.

Interestingly, our results showed that ZAG overexpression also had a remarkable effect on whole-body glucose homeostasis in HFD-induced obese mice, as evidenced by significantly ameliorated glucose tolerance, increased insulin sensitivity and reduced fasting glucose levels. This finding was also observed in *ob/ob* mice, in which ZAG administration significantly decreased blood glucose, ameliorated glucose intolerance and increased insulin sensitivity (Russell and Tisdale, 2010). In addition, studies performed in human beings also reported plasma ZAG levels to be negatively correlated with insulin levels and HOMA-IR (Ceperuelo-Mallafré et al., 2009; Yang et al., 2013), and circulating ZAG levels were lower in prediabetic and type 2 diabetes patients than in the corresponding controls (Yang et al., 2013). Thus, all these findings together with our results suggest that ZAG also regulates glucose metabolism in addition its role in regulating body weight and body fat.

It is well-documented that the metabolic effects of ZAG occur through interaction with β3-AR (Russell et al., 2002; Russell and Tisdale, 2012b), while β3-AR activation can trigger thermogenesis by inducing *UCP* expression in BAT (Himms-Hagen et al., 1994) and skeletal muscle (Yoshida et al., 1998; Yoshitomi et al., 1998). More importantly, recent studies revealed the presence of active brown adipocytes in WAT, also called “beige” or “brite” cells, which resembled white adipocytes in basal conditions but rapidly acquired a brown phenotype and expressed high levels of *UCP1* upon pharmacological activation of β3-AR (Harms and Seale, 2013). Stimulation of BAT activity and/or WAT browning is an important target for the treatment of obesity/insulin resistance (Poher et al., 2015). Studies performed *in vitro* by Sanders et al. showed that ZAG could dose-dependently increase the expression of *UCP-1* in BAT and *UCP-2* expression in myotubes, and this effect was attenuated by the β3-AR antagonist SR59230A (Sanders and Tisdale, 2004). In our present study, we firstly found that the expression of *ZAG* and WAT browning-related genes—*PGC1*α, *PRDM16, CIDEA, PPAR*γ*2*, and *UCP1*—were significantly reduced in the sWAT of obese patients, and there was a strong positive relationship between the mRNA levels of *ZAG* and these WAT browning-related genes. However, the expression of UCP1 in human sWAT could only be detected in mRNA levels and our findings were largely correlatively. Further *in vivo* studies showed that PGC1α mRNA and protein levels in the subcutaneous and visceral WAT of HFD-induced obese mice significantly increased after ZAG treatment, and a strong positive relationship in mRNA levels between *Zag* and *Pgc1*α in subcutaneous WAT was also observed in mice. Previous *in vitro* studies by Balaz et al. found that silencing ZAG gene expression in primary human adipocytes resulted in a 26% reduction in *PGC1a* mRNA expression (Balaz et al., 2014). All these results indicate a close relationship between ZAG and PGC1α expression in sWAT, and PGC1α may play an important role in ZAG action. It is well-known that PGC1α is a key transcriptional coactivator that triggers WAT browning by activating PPAR isoforms (PPARα, PPARβ, PPARγ)—a range of transcription factors associated with UCP1 transcription (Wang et al., 2003) and that it is involved in stimulating mitochondrial biogenesis. Previous studies have shown that β3-AR agonists may stimulate cyclic AMP/protein kinase A, thus leading to activation of PCG-1α as well as ATF-2, then allowing the CRE and PPAR elements of the UCP1 enhancer to be occupied, ultimately causing increased UCP1 expression in BAT (Collins et al., 2004). In our present study, we found that the UCP1-staining in the sWAT of ZAG-treated mice were stronger than those of controls, and the protein levels of UCP1 in the sWAT of ZAG treated mice were significantly increased. In addition, *Ucp1* mRNA expression in visceral WAT also tended to be increased. All these findings suggest that ZAG reduces body fat mass and ameliorates glucose metabolism by activating PGC1α and UCP1 expression in subcutaneous and visceral WAT, then promoting WAT browning in obese mice.

Our present findings also showed that *G6pc* mRNA expression were notably attenuated while *Pgc1a* mRNA expression increased significantly in the liver after 8 weeks of ZAG administration. PGC-1α is known to be an important regulator of gluconeogenesis in the liver. Although PGC-1α has been shown to be involved in the pathogenesis of hepatic insulin resistance, the same molecule also promotes mitochondrial biogenesis and glucose/fatty acid metabolism in peripheral tissues (Liang and Ward, 2006). A great number of studies in a variety of models has shown that the increased hepatic fatty acid disposal has well-documented effects on liver insulin sensitivity (Petersen et al., 2003; Nandi et al., 2004; Savage et al., 2007). Taking all these results together, we could speculate that the effect of ZAG on glycemic control may be realized by directly suppressing hepatic gluconeogenesis as well as by indirectly stimulating hepatic fatty acid disposal via increasing the expression of PGC1a. Additionally, studies performed by Russel et al. found that ZAG ameliorated the glucose metabolism of *ob/ob* mice by increasing the expression of *Glut4* in skeletal muscle (Russell and Tisdale, 2010). However, *Glut4* expression in skeletal muscle showed no significant change in ZAG-treated mice in our present study. One explanation for this discrepancy is that skeletal muscle fibers can be classified as either slow/type I or fast/type II (Edgerton et al., 1975; Schiaffino and Reggiani, 2011), and *Glut4* is expressed more abundantly in slow/type I muscle fiber (Stuart et al., 2009). In our present study, we did not distinguish slow/type I muscle fiber from fast/type II muscle fiber, which may explain the deviation from previously published results.

It has been reported that ZAG-induced reduction of body fat may be realized through its lipolytic effect because incubation of ZAG with adipocytes isolated from adipose tissue has been shown to stimulate glycerol release in a dose-dependent manner (Russell et al., 2004). In line with this result, our previous study found that the expression of hormone-sensitive lipase, a rate-limiting enzyme in lipolysis, increased significantly in eVAT after mice were treated with ZAG (Gong et al., 2009). Our present study further found that serum levels of free fatty acid levels—the products of lipolysis—were significantly increased after ZAG intervention, suggesting enhanced lipolysis after ZAG administration. In addition, our previous study found that ZAG overexpression could downregulate the expression of lipogenic enzymes including fatty acid synthase (FAS), acetyl-CoA carboxylase (ACC), and acyl-coenzyme A: diacylglycerol transferase (DGAT) in the eVAT of HFD-induced obese mice (Gong et al., 2009). All these finding suggest that the effects of ZAG may be realized through multiple actions including inhibiting lipogenesis, stimulating lipolysis, and promoting WAT browning. However, whether these pathways act independently or interact with each other needs to be further investigated.

Regarding the issue of endogenous vs. exogenous ZAG, our previous studies together with others showed that injection of a ZAG expression plasmid in mice could increase serum ZAG levels (Gong et al., 2009; Fan et al., 2017) as well as *Zag* gene expression in eVAT (Gong et al., 2009). In our present study, we found that administration of a ZAG expression plasmid to mice led to an increase in *Zag* expression in pVAT (Figure 5A) as well as a tendency toward an increase in sWAT (Figure 4B). *In vivo* studies performed by Russell et al. also demonstrated that administration of ZAG could increase the expression of *Zag* in epididymal, subcutaneous, and visceral adipose tissues (Russell and Tisdale, 2011). Moreover, the biological effects of ZAG were still maintained for 3 days in the absence of ZAG (Russell and Tisdale, 2011). These findings suggest that exogenous ZAG may first increase serum ZAG levels, then increase ZAG synthesis and secretion in adipose tissue in a positive feedback loop.

The present study is the first to demonstrate that, in addition to decreased ZAG levels in serum, *ZAG* mRNA levels in sWAT were significantly reduced in Chinese patients with morbid obesity. Additionally, the role of ZAG in reducing body weight, fat mass and improving insulin sensitivity in HFD-induced obese mice was found for the first time to be associated with activating WAT browning-related gene expression, especially that of PGC1α and UCP1, in sWAT and pVAT in addition to its role in regulating lipogenesis and lipolysis. Although further investigation is needed on many more questions regarding WAT browning after ZAG treatment, such as mitochondria biogenesis and cell oxygen consumption, this study a promising beginning that leads us to consider the potential role of WAT browning in the action of ZAG.

## Author contributions

ML did molecular biological experiments, analyzed the data and wrote the primary manuscript; HZ designed the experiments and revised the primary manuscript; YD did the animal experiments; HP, NL, LW, HY, and KY collected the clinical materials, serum samples and finished the clinical and biochemical parameters measurements; FG designed the experiment, supervised the whole experiments and revised the primary manuscript.

### Conflict of interest statement

The authors declare that the research was conducted in the absence of any commercial or financial relationships that could be construed as a potential conflict of interest.

## References

[B1] Balaz M. Vician M. Janakova Z. Kurdiova T. Surova M. Imrich R.. (2014). Subcutaneous adipose tissue zinc-α_2_-glycoprotein is associated with adipose tissue and whole-body insulin sensitivity. Obesity 22 1821–1829. 10.1002/oby.20764 24753506

[B2] Bao Y. Bing C. Hunter L. Jenkins J. R. Wabitsch M. Trayhurn P. (2005). Zinc-α_2_-glycoprotein, a lipid mobilizing factor, is expressed and secreted by human (SGBS) adipocytes. FEBS Lett. 579, 41–47. 10.1016/j.febslet.2004.11.042 15620688

[B3] Ceperuelo-Mallafré V. Näf S. Escoté X. Caubet E. Gomez J. M. Miranda M.. (2009). Circulating and adipose tissue gene expression of zinc-α_2_-glycoprotein in obesity: its relationship with adipokine and lipolytic gene markers in subcutaneous and visceral fat. J. Clin. Endocrinol. Metab. 94 5062–5069. 10.1210/jc.2009-0764 19846741

[B4] Collins S. Cao W. Robidoux J. (2004). Learning new tricks from old dogs: β-adrenergic receptors teach new lessons on firing up adipose tissue metabolism. Mol. Endocrinol. 18, 2123–2131. 10.1210/me.2004-0193 15243132

[B5] Edgerton V. R. Smith J. L. Simpson D. R. (1975). Muscle fibre type populations of human leg muscles. Histochem J. 7, 259–266. 10.1007/BF01003594 123895

[B6] Fan G. Qiao Y. Gao S. Guo J. Zhao R. Yang X. (2017). Effects of zinc α_2_ glycoprotein on lipid metabolism of liver in high-fat diet-induced obese mice. Horm. Metab. Res. 49, 793–800. 10.1055/s-0043-118910 28934818

[B7] Fisher F. M. Kleiner S. Douris N. Fox E. C. Mepani R. J. Verdeguer F.. (2012). FGF21 regulates PGC-1α and browning of white adipose tissues in adaptive thermogenesis. Genes. Development. 26, 271–281. 10.1101/gad.177857.111 22302939 PMC3278894

[B8] Garrido-Sánchez L. García-Fuentes E. Fernández-García D. Escoté X. Alcaide J. Perez-Martinez P.. (2012). Zinc-alpha 2-glycoprotein gene expression in adipose tissue is related with insulin resistance and lipolytic genes in morbidly obese patients. PLoS ONE 7:e33264. 10.1371/journal.pone.0033264 22442679 PMC3307730

[B9] Gong F. Zhang S. Deng J. Zhu H. Pan H. Li N. (2009). Zinc-a2-glycoprotein is involved in regulation of body weight through inhibition of lipogenic enzymes in adipose tissue. Int. J. Obes. 33, 1023–1030. 10.1038/ijo.2009.141 19621019

[B10] Harms M. Seale P. (2013). Brown and beige fat: development, function and therapeutic potential. Nat. Med. 10, 1252–1263. 10.1038/nm.3361 24100998

[B11] Himms-Hagen J. Cui J. Danforth E. Jr. Taatjes D. J. Lang S. S. Waters B. L.. (1994). Effect of CL-316,243, a thermogenic β 3-agonist, on energy balance and brown and white adipose tissues in rats. Am. J. Physiol. 266, R1371–R1382. 10.1152/ajpregu.1994.266.4.R1371 7910436

[B12] Hirai K. Hussey H. J. Barber M. D. Price S. A. Tisdale M. J. (1998). Biological evaluation of a lipid-mobilizing factor isolated from the urine of cancer patients. Cancer. Res. 58, 2359–2365. 9622075

[B13] Kajimura S. Spiegelman B. M. Seale P. (2015). Brown and beige fat: physiological roles beyond heat generation. Cell Metab. 22, 546–559. 10.1016/j.cmet.2015.09.007 26445512 PMC4613812

[B14] Liang H. Ward W. F. (2006). PGC-1alpha: a key regulator of energy metabolism. Adv. Physiol. Educ. 30, 145–151. 10.1152/advan.00052.2006 17108241

[B15] Livak K. J. Schmittgen T. D. (2001). Analysis of relative gene expression data using real-time quantitative PCR and the 2(-Delta Delta C(T)) Method. Methods 4, 402–408. 10.1006/meth.2001.1262 11846609

[B16] Mracek T. Ding Q. Tzanavari T. Kos K. Pinkney J. Wilding J.. (2010). The adipokine zinc-α_2_-glycoprotein (ZAG) is downregulated with fat mass expansion in obesity. Clin. Endocrinol. 72, 334–341. 10.1111/j.1365-2265.2009.03658.x 19549246

[B17] Nandi A. Kitamura Y. Kahn C. R. Accili D. (2004). Mouse models of insulin resistance. Physiol. Rev. 84, 623–647. 10.1152/physrev.00032.2003 15044684

[B18] O'Neill S. O'Driscoll L. (2015). Metabolic syndrome: a closer look at the growing epidemic and its associated pathologies. Obes. Rev. 1, 1–12. 10.1111/obr.12229 25407540

[B19] Petersen K. F. Befroy D. Dufour S. Dziura J. Ariyan C. Rothman D. L.. (2003). Mitochondrial dysfunction in the elderly: possible role in insulin resistance. Science 300, 1140–1142. 10.1126/science.1082889 12750520 PMC3004429

[B20] Poher A. L. Altirriba J. Veyrat-Durebex C. Rohner-Jeanrenaud F. (2015). Brown adipose tissue activity as a target for the treatment of obesity/insulin resistance. Front. Physiol. 6:4. 10.3389/fphys.2015.00004 25688211 PMC4311629

[B21] Rolli V. Radosavljevic M. Astier V. Macquin C. Castan-Laurell I. Visentin V.. (2007). Lipolysis is altered in MHC class I zinc-alpha (2)-glycoprotein deficient mice. FEBS. Lett. 581, 394–400. 10.1016/j.febslet.2006.12.047 17234189

[B22] Russell S. T. Tisdale M. J. (2010). Antidiabetic properties of zinc-a2-glycoprotein in ob/ob mice. Endocrinology 151, 948–957. 10.1210/en.2009-0827 20032055

[B23] Russell S. T. Tisdale M. J. (2011). Studies on the antiobesity effect of zinc-a2-glycoprotein in the ob/ob mouse. Int. J. Obes. 35, 345–354. 10.1038/ijo.2010.150 20697416

[B24] Russell S. T. Tisdale M. J. (2012a). Role of β-adrenergic receptors in the anti-obesity and anti-diabetic effects of zinc-α_2_-glycoprotien (ZAG). Biochim. Biophys. Acta. 1821, 590–599. 10.1016/j.bbalip.2011.12.003 22227600

[B25] Russell S. T. Tisdale M. J. (2012b). Role of β-adrenergic receptors in the oral activity of zinc-α_2_-glycoprotein (ZAG). Endocrinology 153, 4696–4704. 10.1210/en.2012-1260 22903615

[B26] Russell S. T. Hirai K. Tisdale M. J. (2002). Role of β3-adrenergic receptors in the action of a tumour lipid mobilizing factor. Br. J.Cancer. 86, 424–428. 10.1038/sj.bjc.6600086 11875710 PMC2375201

[B27] Russell S. T. Zimmerman T. P. Domin B. A. Tisdale M. J. (2004). Induction of lipolysis *in vitro* and loss of body fat *in vivo* by zinc-α_2_-glycoprotein. Biochim. Biophys. Acta. 1636, 59–68. 10.1016/j.bbalip.2003.12.004 14984739

[B28] Sanders P. M. Tisdale M. J. (2004). Effect of zinc-α_2_-glycoprotein (ZAG) on expression of uncoupling proteins in skeletal muscle and adipose tissue. Cancer. Lett. 212, 71–81. 10.1016/j.canlet.2004.03.021 15246563

[B29] Savage D. B. Petersen K. F. Shulman G. I. (2007). Disordered lipid metabolism and the pathogenesis of insulin resistance. Physiol. Rev. 87, 507–520. 10.1152/physrev.00024.2006 17429039 PMC2995548

[B30] Schiaffino S. Reggiani C. (2011). Fiber types in mammalian skeletal muscles. Physiol. Rev. 91, 1447–1531. 10.1152/physrev.00031.2010 22013216

[B31] Selva D. M. Lecube A. Hernandez C. Baena J. A. Fort J. M. Simo R. (2009). Lower zinc-α_2_-glycoprotein production by adipose tissue and liver in obese patients unrelated to insulin resistance. J. Clin. Endocrinol. Metab. 94, 4499–4507. 10.1210/jc.2009-0758 19622624

[B32] Stuart C. A. Howell M. E. Zhang Y. Yin D. (2009). Insulin-stimulated translocation of glucose transporter (GLUT) 12 parallels that of GLUT4 in normal muscle. J. Clin. Endocrinol. Metab. 94, 3535–3542. 10.1210/jc.2009-0162 19549745 PMC2741719

[B33] Todorov P. T. McDevitt T. M. Meyer D. J. Ueyama H. Ohkubo I. Tisdale M. J. (1998). Purification and characterization of a tumor lipid-mobilizing factor. Cancer. Res. 58, 2353–2358. 9622074

[B34] Wallace T. M. Levy J. C. Matthews D. R. (2004). Use and abuse of HOMA modeling. Diabetes Care 27, 1487–1495. 10.2337/diacare.27.6.1487 15161807

[B35] Wang Y. Lee C. Tiep S. Yu R. Ham J. Kang H.. (2003). Peroxisome-proliferator- activated receptor delta activates fat metabolism to prevent obesity. Cell 113, 159–170. 10.1016/S0092-8674(03)00269-1 12705865

[B36] Yang M. Liu R. Li S. Luo Y. Zhang Y. Zhang L.. (2013). Zinc- 2-Glycoprotein is associated with insulin resistance in humans and is regulated by hyperglycemia, hyperinsulinemia, or liraglutide administration: cross-sectional and interventional studies in normal subjects, insulin-resistant subjects, and subjects with newly diagnosed diabetes. Diabetes Care 36, 1074–1082. 10.2337/dc12-0940 23275352 PMC3631846

[B37] Yoshida T. Umekawa T. Kumamoto K. Sakane N. Kogure A. Kondo M.. (1998). β 3-Adrenergic agonist induces a functionally active uncoupling protein in fat and slow-twitch muscle fibers. Am. J. Physiol. 274, E469–E475. 9530130 10.1152/ajpendo.1998.274.3.E469

[B38] Yoshitomi H. Yamazaki K. Abe S. Tanaka I. (1998). Differential regulation of mouse uncoupling proteins among brown adipose tissue, white adipose tissue, and skeletal muscle in chronic β 3 adrenergic receptor agonist treatment. Biochem. Biophys. Res. Commun. 253, 85–91. 10.1006/bbrc.1998.9746 9875224

[B39] Zhu H. Dong C. Pan H. Ping X. Li N. Dai Y.. (2012). rs4215 SNP in zinc-α_2_-glycoprotein gene is associated with obesity in Chinese north Han population. Gene 500, 211–215. 10.1016/j.gene.2012.03.020 22425975

[B40] Zhu H. Pan H. Zhang X. Li N. Wang L. Yang H.. (2015). The effect of myostatin on proliferation and lipid accumulation in 3T3-L1 preadipocytes. J. Mol. Endocrinol. 54, 217–226. 10.1530/JME-15-0038 25878062

[B41] Zhu H. Wang X. Pan H. Dai Y. Li N. Wang L.. (2016). The mechanism by which safflower yellow decreases body fat mass and improves insulin sensitivity in HFD-induced obese mice. Front. Pharmacol. 7:127. 10.3389/fphar.2016.00127 27242533 PMC4876777

