# Supplementary material for: Zinc-α2-Glycoprotein Is Associated with Obesity in Chinese People and HFD-Induced Obese Mice
Source: Front Physiol. 2018 Feb 7;9:62. doi: 10.3389/fphys.2018.00062 (PMC5808341; doi:10.3389/fphys.2018.00062)
Supplement: Supplementary file 1 [file Table1.DOCX]

| **Supplementary Table 1. Primers sequences used for RT-qPCR in human adipose tissue** | | |
| --- | --- | --- |
| Gene | Forward primer | Reverse primer |
| *β-actin* | ACTCTTCCAGCCTTCCTT | GATCTTCATTGTGCTGGG |
| *ZAG* | GAGACCGAAGACTGGGATG | CAAGGAGGGATGATTATTTATTAGC |
| *UCP1* | TGGTGTCGGCTCTTATCG | CGTTGGTCCTTCGTTAGTG |
| *PGC1α* | GCTTTCTGGGTGGACTCAAGT | GAGGGCAATCCGTCTTCATCC |
| *CIDEA* | GATGCCCTCGTCATCGCTAC | GCGTGTTGTCTCCCAAGGTC |
| *PRDM16* | CGAGGCCCCTGTCTACATTC | GCTCCCATCCGAAGTCTGTC |
| *PPARγ2* | GGGATCAGCTCCGTGGATCT | TGCACTTTGGTACTCTTGAAGTT |

Abbreviations: ZAG, zinc-α2-glycoprotein; UCP1, uncoupling protein 1; PGC1α, peroxisome proliferator-activated receptor gamma coactivator 1 alpha; CIDEA, cell death-inducing DFFA-like effector α; PRDM16, PR/SET domain 16; PPARγ2, peroxisome proliferator activated receptor gamma 2.
