# Supplementary material for: Zinc-α2-Glycoprotein Is Associated with Obesity in Chinese People and HFD-Induced Obese Mice
Source: Front Physiol. 2018 Feb 7;9:62. doi: 10.3389/fphys.2018.00062 (PMC5808341; doi:10.3389/fphys.2018.00062)
Supplement: Supplementary file 2 [file Table2.DOCX]

| **Supplementary Table 2. Primers sequences used for RT-qPCR in mouse adipose**  **tissue, liver and skeletal muscle** | | |
| --- | --- | --- |
| Gene | Forward primer | Reverse primer |
| *Ppia* | GCTGGACCAAACACAAACGG | TCCTGGACCCAAAACGCTC |
| *β-actin* | CCTGAACCCTAAGGCCAACC | GTACATGGCTGGGGTGTTGA |
| *Pgc1α* | TTTACGCAGGTCGAACGAAAC | GTGGAAGCAGGGTCAAAATCG |
| *Zag*  *Ucp1* | CAAGGACACTACAGGGTCTCA  TCTCTGCCAGGACAGTACCCAA | AATCCTCTCCGTCGTAGGCAT  GAGTCGCAGAAAAGAAGCCACAA |
| *Glut4* | GTTGGTCTCGGTGCTCTTAGTAG | CGATGGAGACATAGCTCATGGC |
| *Irs1* | AAGACGCTCCAGTGAGGATT | AGGAGGATTTGCTGAGGTCATT |
| *Gys1* | GAACGCAGTGCTTTTCGAGG | CCAGATAGTAGTTGTCACCCCAT |
| *Gys2*  *G6pc*  *Pck1* | ACCAAGGCCAAAACGACAG  CGACTCGCTATCTCCAAGTGA  AGCATTCAACGCCAGGTTC | GGGCTCACATTGTTCTACTTGA  GGGCGTTGTCCAAACAGAAT  CGAGTCTGTCAGTTCAATACCAA |

Abbreviations: Ppia, peptidylprolyl isomerase A; Pgc1α, peroxisome proliferator-activated receptor gamma coactivator 1 alpha; Zag, zinc-α2-glycoprotein; Ucp1, uncoupling protein 1; Glut4, glucose transporter type 4; Irs1, insulin receptor substrate 1; Gys1, glycogen synthase 1; Gys2, glycogen synthase 2; G6pc, glucose-6-phosphatase, catalytic; Pck1, phosphoenolpyruvate carboxykinase 1, cytosolic.
